# A SSR-based composite genetic linkage map for the cultivated peanut (*Arachis hypogaea *L.) genome

**DOI:** 10.1186/1471-2229-10-17

**Published:** 2010-01-27

**Authors:** Yanbin Hong, Xiaoping Chen, Xuanqiang Liang, Haiyan Liu, Guiyuan Zhou, Shaoxiong Li, Shijie Wen, C Corley Holbrook, Baozhu Guo

**Affiliations:** 1 Guangdong Academy of Agricultural Sciences, Crops Research Institute, Guangzhou, PR China; 2 US Department of Agriculture, Agricultural Research Service, Crop Protection and Management Research Unit, Tifton, GA 31793, USA; 3 University of Georgia, Department of Plant Pathology, Tifton, GA 31793, USA; 4 US Department of Agriculture, Agricultural Research Service, Crop Genetics and Breeding Research Unit, Tifton, GA 31793, USA

## Abstract

**Background:**

The construction of genetic linkage maps for cultivated peanut (*Arachis hypogaea *L.) has and continues to be an important research goal to facilitate quantitative trait locus (QTL) analysis and gene tagging for use in a marker-assisted selection in breeding. Even though a few maps have been developed, they were constructed using diploid or interspecific tetraploid populations. The most recently published intra-specific map was constructed from the cross of cultivated peanuts, in which only 135 simple sequence repeat (SSR) markers were sparsely populated in 22 linkage groups. The more detailed linkage map with sufficient markers is necessary to be feasible for QTL identification and marker-assisted selection. The objective of this study was to construct a genetic linkage map of cultivated peanut using simple sequence repeat (SSR) markers derived primarily from peanut genomic sequences, expressed sequence tags (ESTs), and by "data mining" sequences released in GenBank.

**Results:**

Three recombinant inbred lines (RILs) populations were constructed from three crosses with one common female parental line Yueyou 13, a high yielding Spanish market type. The four parents were screened with 1044 primer pairs designed to amplify SSRs and 901 primer pairs produced clear PCR products. Of the 901 primer pairs, 146, 124 and 64 primer pairs (markers) were polymorphic in these populations, respectively, and used in genotyping these RIL populations. Individual linkage maps were constructed from each of the three populations and a composite map based on 93 common loci were created using JoinMap. The composite linkage maps consist of 22 composite linkage groups (LG) with 175 SSR markers (including 47 SSRs on the published AA genome maps), representing the 20 chromosomes of *A. hypogaea*. The total composite map length is 885.4 cM, with an average marker density of 5.8 cM. Segregation distortion in the 3 populations was 23.0%, 13.5% and 7.8% of the markers, respectively. These distorted loci tended to cluster on LG1, LG3, LG4 and LG5. There were only 15 EST-SSR markers mapped due to low polymorphism. By comparison, there were potential synteny, collinear order of some markers and conservation of collinear linkage groups among the maps and with the AA genome but not fully conservative.

**Conclusion:**

A composite linkage map was constructed from three individual mapping populations with 175 SSR markers in 22 composite linkage groups. This composite genetic linkage map is among the first "true" tetraploid peanut maps produced. This map also consists of 47 SSRs that have been used in the published AA genome maps, and could be used in comparative mapping studies. The primers described in this study are PCR-based markers, which are easy to share for genetic mapping in peanuts. All 1044 primer pairs are provided as additional files and the three RIL populations will be made available to public upon request for quantitative trait loci (QTL) analysis and linkage map improvement.

## Background

Legumes are a diverse and important family of angiosperms. With more than 650 genera and 18,000 species, legumes are the third largest family of higher plants and are second only to grasses in agriculture [1]. Peanut or groundnut is one of the major economically-important legumes that are widely grown in China, India, United States, and many countries in South America and Africa. Peanut is important for its ability to grow in semi-arid environments with relatively low inputs of costly resources such as chemical fertilizers. Peanut is also a major source of protein and vegetable oil for human nutrition on a global basis. On average of the years, 2001 to 2003, peanut was grown on 22.5 million hectares with a total global production of 32 million metric tons http://www.nass.usda.gov/Publications/Ag_Statistics/2004. In the same period, the U.S. peanut crop averaged 598 thousand hectares with a total production on average of 1.6 million metric tons (2.2 million metric tons for 2005) concentrating in nine Southern States, including Alabama, Florida, Georgia, New Mexico, North Carolina, Oklahoma, South Carolina, Texas, and Virginia.

There is considerable variation in *Arachis hypogaea *subspecies *hypogaea *and *fastigiata*, which are further classified into runner, Virginia, Spanish, and Valencia market types [2]. Most cultivated peanuts belong to Spanish and runner types. They exhibit genetically-determined variation for a number of botanical and agronomical traits including branching and flowering habits, seed dormancy, and maturation time. However, cultivated peanut is an allotetraploid (2n = 4× = 40), with little polymorphism at the molecular level [3-7] as indicated by using traditional markers such as RAPD and RFLP. Pairing in *A. hypogaea *is generally bivalent, with occasional higher-order associations found in crosses among different market types [8]. Cultivated peanut is considered to have originated from a single recent polyploidization event [9,10], unlike many other natural polyploidy species for which polyploidization events have been identified. The most likely wild diploid progenitors for cultivated peanut are *A. duranensis *(the A genome) and *A. ipaensis *(the B genome) [9]. Even though peanut is an important crop economically and nutritionally, narrow genetic diversity and a deficiency of polymorphic DNA markers in the public database have hindered genetic mapping and the application of molecular breeding in cultivated peanut. Nevertheless, the peanut research community still lacks adequate tools and resources for peanut genetic and genomic research and breeding, and therefore, for expanding our basic knowledge of the genetic control of complex traits.

A genetic map constructed from a population segregating for a trait of interest is required for QTL (quantitative trait loci) identification. Peanut exhibits a considerable amount of variability for morphological traits and for resistance to insects and diseases. However, a more detailed linkage map of all chromosomes and with sufficient markers is necessary to be feasible for QTL identification and marker-assisted selection. There is one RFLP map of diploid peanut developed from the interspecific hybridization (F_2 _population) of two related diploid species with AA genome (*A. stenosperma *and *A. cardenasii*) of peanut with 11 linkage groups [11]. There is another RFLP map of a synthetic interspecific tetraploid population {[*A. batizocoi *× (*A. cardenasii *× *A. diogoi*)]^4× ^× *A. hypogaea*} using 78 BC_1 _population with 23 linkage groups [12]. Because of the complex pedigree, this map is complicated and difficult to use in terms of extraction of useful information. Moretzsohn *et al*. [13] published a SSR-based linkage map (F_2 _population) for the AA genome of diploid wild peanut (*A. duranensis *and *A. stenosperm*a) with 170 SSRs and 11 linkage groups, and an advanced version of same map has been published with 369 markers, including 188 microsatellites, 80 legume anchor markers, 46 AFLPs, 32 NBS profiling, 17 SNP, 4 RGA-RFLP and 2 SCAR markers, mapped into 10 linkage groups by Leal-Bertioli et al. [14]. Hong *et al*. [15] published a SSR-based map (F_4:6 _RILs) for cultivated peanuts with 131 SSR and 20 linkage groups. Varshney et al. [16] published the most recent intra-specific map constructed from the cross of cultivated peanuts, in which 135 SSR markers were sparsely populated in 22 linkage groups. Foncéka et al. [17] published a SSR-based map using 88 individuals of the BC_1_F_1 _population of [Fleur 11 × (*A. ipaënsis *× *A. duranensis*)^4×^, and 298 loci were mapped in 21 linkage groups (LGs). Nevertheless, the application of biotechnology to the improvement of the allotetraploid cultivated peanut has been hampered by an inability to visualize genetic variation and by lacking a road-map.

Simple sequence repeat (SSR) markers are PCR (polymerase chain reaction) based markers that are reproducible and detect co-dominant multi-allelic loci [18]. Recent studies have shown that SSRs can detect more polymorphism in cultivated peanut than RFLP, RAPD and AFLP [19-21]. In recent years, a large number of SSR markers for peanut have been developed from genomic DNA libraries and expressed sequence tags (EST) with the goal of providing sufficient sequence resources for developing a critical mass of DNA markers for the community [22,23], making it feasible to use SSRs to construct a genetic linkage map.

The various peanut maps constructed to date have few markers in common and it is impossible to conduct comparative mapping. SSR-based markers provide a set of easily shared markers that can be used to unify and cross reference established genetic maps. The SSRs developed in this study will provide an important resource for genetic mapping and marker-assisted selection, as well as for comparative genetic studies between cultivated and wild peanuts (AA genome) and other legumes. Therefore, the objective of this study was to construct a SSR-based composite reference map for cultivated peanut using public available SSR sources [13,19,24-28], GenBank [29] and newly developed ESTs [22,23]. The SSR primers and the RIL populations used in this study will be made available to other researchers for comparative mapping, QTL analysis and map improvement (Additional files 1 and 2).

## Results

Three mapping populations of recombinant inbred lines (RILs) of cultivated peanuts were derived from three crosses with a common female parent, Yueyou 13 (Y13), a Spanish bunch type with high yield. The populations were advanced to the F_4_, using single seed descent. Individual plants were harvested and progeny rows were grown to produce the F_4:6 _RIL populations.

**Figure 1:**
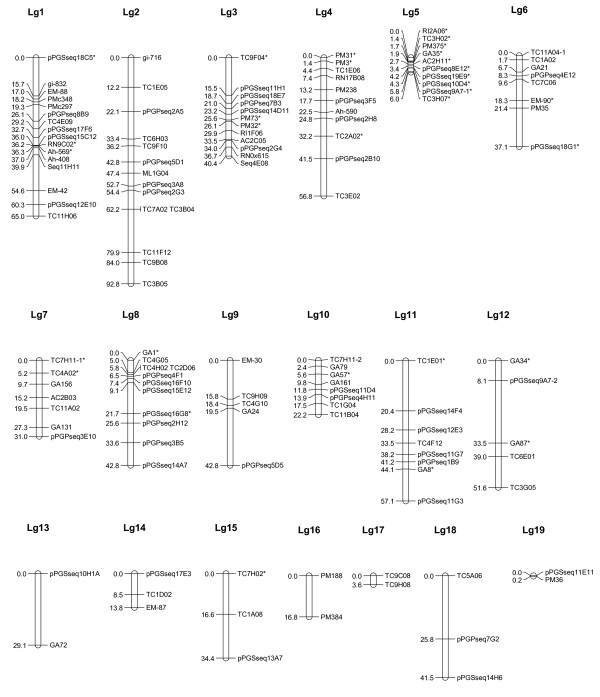
**Genetic linkage groups based on population Y13Zh (Yueyou 13 × Zhenzhuhei)**. RIL (recombinant inbred line) population Y13Zh consisted of 142 lines, derived from a cross made from the female parent Yueyou13, a Spanish type with high yield, and male parent Zhenzhuhei, a Virginia type with dark purple testa and high protein (32.4%) content. Linkage analysis was performed with JoinMap using a minimum LOD score of 3.0 and linkage maps were drawn using MapChart for Windows. Underlined markers were EST-SSRs. Markers that showed significant distortions from 1:1 segregation are indicated by *. Markers that amplified two loci are designated by an Arabic number (-1 or -2) appended to the locus name to distinguish the two loci 1 and 2 after the marker name.

**Figure 2:**
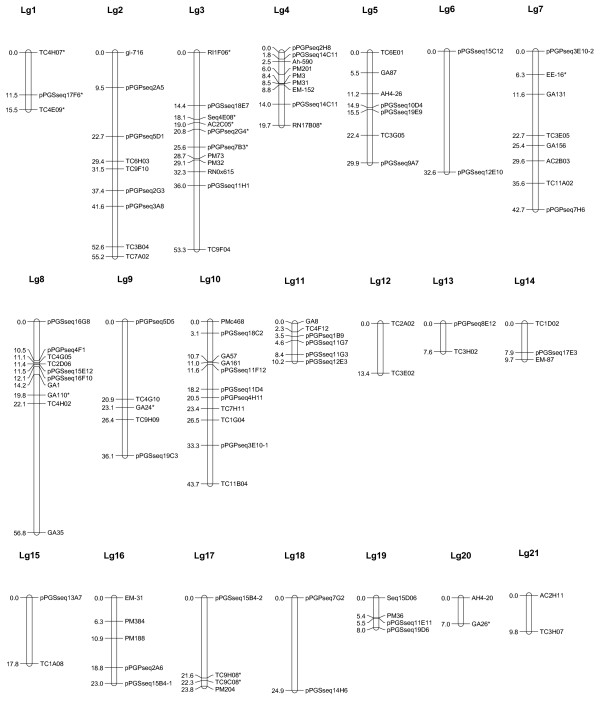
**Genetic linkage groups based on population Y13Fu (Yueyou 13 × Fu 95-5)**. RIL (recombinant inbred line) population Y13Fu consisted of 84 lines, derived from a cross made from the female parent Yueyou13, a Spanish type with high yield, and male parent Fu 95-5, a Spanish type with high oil content (56.2%). Linkage analysis was performed with JoinMap using a minimum LOD score of 3.0 and linkage maps were drawn using MapChart for Windows. Underlined markers were EST-SSRs. Markers that showed significant distortions from 1:1 segregation are indicated by *. Markers that amplified two loci are designated by an Arabic number (-1 or -2) appended to the locus name to distinguish the two loci 1 and 2 after the marker name.

**Figure 3:**
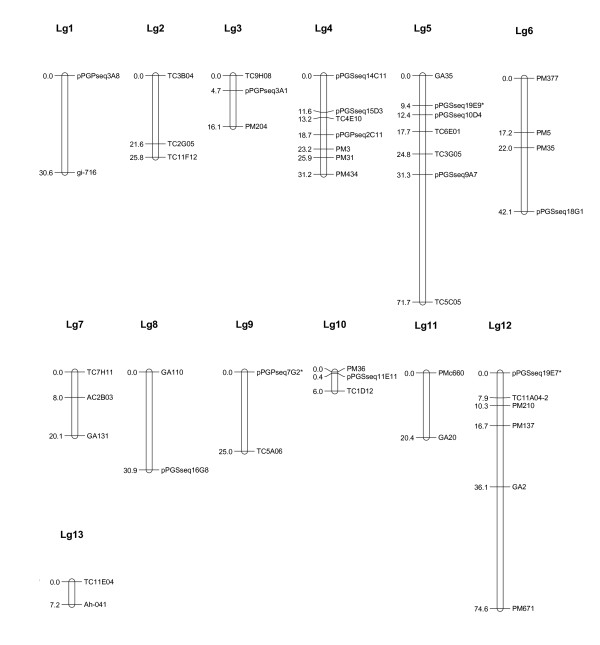
**Genetic linkage groups based on population Y13J11 (Yueyou 13 × J11)**. RIL (recombinant inbred line) population Y13J11 consisted of 136 lines, derived from the female parent Yueyou13, a Spanish type with high yield, and male parent J11, a Spanish type with reported resistance to *Aspergillus flavus *and aflatoxin contamination, by single seed descent from F4 to F6 generation and consisted of 136 individual lines. Linkage analysis was performed with JoinMap using a minimum LOD score of 3.0 and linkage maps were drawn using MapChart for Windows. Underlined markers were EST-SSRs. Markers that showed significant distortions from 1:1 segregation are indicated by *. Markers that amplified two loci are designated by an Arabic number (-1 or -2) appended to the locus name to distinguish the two loci 1 and 2 after the marker name.

**Figure 4:**
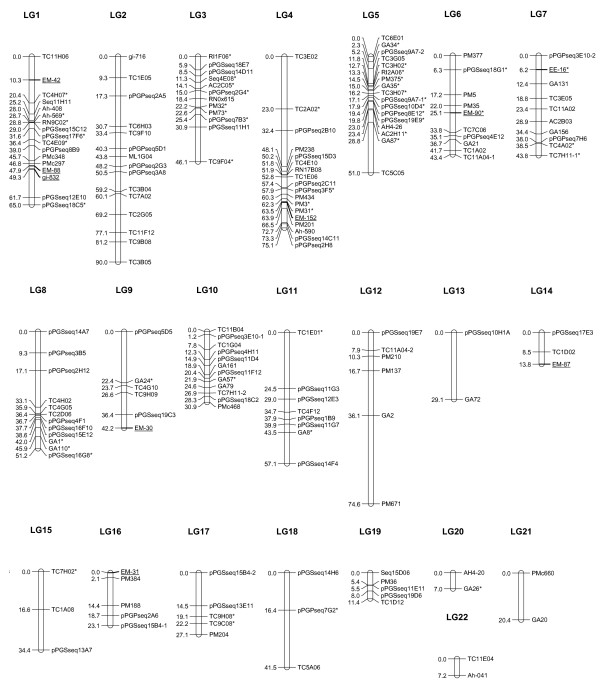
**A SSR-based composite linkage map of *Arachis hypogaea *based on three RIL populations**. Based on the maps of three RIL populations, a composite map was constructed. The composite linkage maps consist of 22 composite linkage groups with 175 SSR marker loci. Linkage analysis was performed with JoinMap using a minimum LOD score of 3.0 and linkage maps were drawn using MapChart for Windows. Underlined markers were EST-SSRs. Markers that showed significant distortions from 1:1 segregation are indicated by *. Markers that amplified two loci are designated by an Arabic number (-1 or -2) appended to the locus name to distinguish the two loci 1 and 2 after the marker name.

A total of 1044 SSR primer pairs were collected and designed to amplify SSR markers in these parental lines, including 652 genomic-SSRs and 392 EST-SSRs (refer to Additional file 1). Among the screened SSRs, 143 primer pairs (97 genomic-SSRs and 46 EST-SSRs) did not produce clear amplification products, and were not included in the RILs genotype screening. The remaining 901 primer pairs were used in polymorphism screening and genotyping among the parental lines and RILs. A total of 192 SSR markers detected polymorphism in at least one mapping population. There were 146, 124, and 64 polymorphic SSRs in the Y13Zh, Y13Fu, and Y13J11 populations, respectively. In this study, markers derived from genomic DNA were more polymorphic than markers developed from ESTs; the percentages of genomic SSRs displaying polymorphisms were 23.6%, 20.9%, and 11.4% in these three populations, respectively, whereas only 4.3%, 2.3% and 0.3% of EST-SSRs showed polymorphism in the corresponding populations. The 192 polymorphic SSR primer pairs amplified 197 segregating loci, of which 5 primer pairs (pPGSseq9A7, TC11A04, TC7H11, pPGPseq3E10, and pPGSseq15B4) detected duplicated loci, which are designated by an Arabic number (-1 or -2) appended to the locus name to distinguish the two loci (Figures 1, 2, 3, and 4). Locus duplication was inferred by the fact that the two loci amplified with the same primer pair was mapped to different locations or linkage groups (representing A genome or B genome).

### Linkage groups from Population Y13Zh (Figure 1)

For population Y13Zh, 146 SSR markers, detected 148 loci, were scored and used for the construction of the linkage groups (Lg, using the low case to distinguish with the upper case of the composite map) by JoinMap software [30]. The markers were assigned to linkage groups at a LOD (logarithm of odds ratio) threshold of 3. The genetic map derived from this population contained 132 loci on 19 linkage groups (Figure 1) spanning a total genetic distance of 684.9 cM with 16 markers remaining unlinked. The length of each linkage group was varying from 0.2 cM (Lg19) to 92.8 cM (Lg2) and each had 2 to 16 markers. This population Y13Zh was also used for map construction by Hong *et al*. [15] resulting in 131 SSR loci on 20 linkage groups. We have analyzed the original genotyping data and improved the linkage groups that have been included in the composite map (Additional file 2; Figure 4).

Thirty-four loci showed segregation distortion in this population, 31 of which were distributed on 11 linkage groups (Figure 1). The number of markers showing segregation distortion varied from 0 to 10 per linkage group. Fifty-eight percent (58%) of the distorted loci were in favor of the alleles of parental line Y13. The most extreme example of segregation distortion was found on Lg5 where all ten loci showed segregation distortion (Figure 1). Lg5 showed an average skewed ratio of 10:132 instead of 1:1 over its entire length, severely favoring alleles from parental line Zh.

### Linkage groups from Population Y13Fu (Figure 2)

A total of 124 SSR polymorphic markers, detected 126 loci that were segregating in population Y13Fu. A linkage map was constructed at a LOD threshold of 3 or higher. There were 109 loci mapped into 21 linkage groups (Lg) (Figure 2), spanning a total genetic distance of 540.69 cM with 17 markers remaining unlinked. On average, this map had 2 to 11 markers on each Lg and the length of each linkage group varied from 7.0 cM (Lg20) to 56.8 cM (Lg8) (Figure 2).

Seventeen loci showed segregation distortion in this population and 15 were assigned on individual linkage group. There were 4 loci in favor of Y13 alleles and 11 in favor of alleles of parental line Fu. The 15 distorted loci were distributed on 7 linkage groups with 5 loci distributed on Lg3, 3 in Lg1, and 2 in Lg17. The remaining loci were distributed on Lg4, Lg8, Lg7, Lg9 and Lg20 (Figure 2).

### Linkage groups from Population Y13J11 (Figure 3)

A total of 64 markers were segregating in population Y13J11. A scaffold map was obtained at a LOD of 3 and higher. Forty-six markers were assigned into 13 linkage groups (Figure 3) and 18 markers remained unlinked. Seven markers were mapped onto Lg5 and Lg4. Six loci were mapped onto Lg12, 4 loci were mapped to Lg6, and 3 loci were mapped to each of Lg2, Lg3, Lg7 and Lg10. The remaining 10 markers were mapped onto 5 linkage groups. There were 5 markers that showed segregation distortion in this population. Two of them were mapped on Lg5 and Lg9. The other three distorted markers could not be assigned onto any linkage group. The length of the map was shorter than the other two maps. It covered 401.7 cM.

### Composite genetic linkage map (Figure 4)

On the basis of these three maps developed from three RIL populations, a composite map was constructed (Figure 4). To construct a composite map, linkage groups in the individual maps with common markers were assigned onto an integrated linkage group. Therefore, the composite maps with 22 linkage groups (LG) were established, of which 17 derived from integration of linkage groups in two or three individual maps. The summary of the composite linkage map is presented in Table 1. This composite tetraploid map consists of 22 composite linkage groups with 175 SSR marker loci including 160 genomic-SSRs and 15 EST-SSRs, covering 885.4 cM of total map distance (Figure 4). The mean interval between adjacent markers is 5.79 cM, and 85% of the intervals between adjacent SSR markers were smaller than 10 cM. Eight intervals (5%) between adjacent markers were between 20 and 40 cM. Among the 175 marker loci, 47 loci with distorted segregation were mapped on the composite maps, and the majority were mapped on LG1 (12.8%), LG3 (17.0%), LG4 (8.5%) and LG5 (25.5%).

**Table 1:** Summary of the "composite" *Arachis hypogaea *genetic linage map

Composite linkage group	Emerging linkage group from individual population			Number of distorted markers	Number of markers	Length (cM)
	**Y13Zh**	**Y13Fu**	**Y13J11**			

LG1	Lg1	Lg1, Lg6		6	17	65.0
LG2	Lg2	Lg2	Lg2, Lg1	0	15	90.0
LG3	Lg3	Lg3		8	12	46.1
LG4	Lg4	Lg4, Lg12	Lg4	4	18	75.1
LG5	Lg5, Lg12	Lg5, Lg13, Lg21	Lg5	12	17	51.0
LG6	Lg6		Lg6	2	10	43.4
LG7	Lg7	Lg7	Lg7	3	10	43.8
LG8	Lg8	Lg8	Lg8	3	12	51.2
LG9	Lg9	Lg9		1	6	42.2
LG10	Lg10	Lg10		1	12	30.9
LG11	Lg11	Lg11		2	8	57.1
LG12			Lg12	1	6	74.6
LG13	Lg13			0	2	29.1
LG14	Lg14	Lg14		0	3	13.8
LG15	L15	Lg15		1	3	34.4
LG16	Lg16	Lg16		0	5	23.1
LG17	Lg17	Lg17	Lg3	2	5	27.1
LG18	Lg18	Lg18	Lg9	1	3	41.5
LG19	Lg19	Lg19	Lg10	0	5	11.4
LG20		Lg20		1	2	7.0

LG21			Lg11	0	2	20.4
LG22			Lg13	0	2	7.2

Total				50	175	885.4

The segregation distortion was 22.8%, 13.6% and 8.5% of the polymorphic markers in these three populations. There were only 15 EST-SSRs mapped due to low polymorphism. A total of 93 markers were in common in at least two of the three mapping populations. Seventeen markers were genotyped in all three populations. In addition, there are 68 more common markers between populations Y13Zh and Y13Fu; 5 more markers between populations Y13Zh and Y13J11, and 3 more SSR markers between populations Y13Fu and Y13J11. Based on the common markers and the comparison between individual maps, the majority of the linkage groups were consistent among the individual maps with few exceptions (Additional file 2). For example, the 7 markers on Lg4 (Figure 1) in the population Y13Zh were placed on two Lgs in the population Y13Fu (5 markers on Lg4 and 2 markers on Lg12) (Figure 2). The markers on Lg5 in population Y13Zh (Figure 1) distributed in 3 Lgs (Lg5, Lg13, and Lg21) in population Y13Fu (Figure 2). The listed inconsistencies may be due to the low density of this linkage map as showing the markers derived from the same linkage group in one population were assigned into different linkage groups in another population (Additional file 1 and 2).

### Comparison of the composite tetraploid peanut map to the AA diploid peanut map

Forty-seven SSRs mapped previously on the AA genome of diploid wild peanut (*A. duranensis *× *A. stenosperm*a) [13] were mapped on the current tetraploid cultivated peanut genetic map (Table 2 and Figure 4). Moretzsohn *et al*. [13] developed a SSR-based linkage map with 170 SSRs and 11 linkage groups. There were 9 sets of SSRs on the AA diploid map were identified corresponding to 11 LGs on the tetraploid map (Table 2, Figure 4), and the largest set of SSR marker common to both maps were 6 that were conservative and collinear in each linkage group, which were mapped on the tetraploid LG4 and AA diploid Group 3. Four SSRs mapping to Group 4 were placed on LG1. Interestingly LG2 has 4 SSRs mapped on Group 8 and 3 SSRs mapped on Group 11, indicating that LG2 involves Groups 8 and 11. There were 7 common markers on Group 2 that were placed on LG3 (3 SSRs), LG7 (2 markers), and one each on LG10 and LG11, suggesting that LGs 3, 7,10 and 11 may belong to one linkage group. Tetraploid LG5 has one marker from Group 1 (TC3H02), two SSRs from Group 5 and two from Group 6, but 3 SSRs mapped on Group 6 were also placed on LG6, giving evidence that LG5 and LG6 may belong to one linkage group or *vice versa*. Another interesting comparison is that 3 SSRs mapped on Group 7 were placed on LG9 (TC4G10) and LG17 (TC9H08 and PM204), supporting that LG9 and LG17 should be one linkage group. Therefore, these SSR markers could provide a set of easily shared markers that can be used to cross reference the tetraploid map to SSR-based AA genome map. On the basis of these common SSR markers, the conservation of collinear linkage groups among these three maps and the composite map and the AA genome wild progenitor (Table 2) could be determined, however, marker order was not fully conserved.

**Table 2:** Summary of comparative information between the tetraploid cultivated peanut (AABB) map and the AA dipoid wild peanut map (Moretzsohn et al. 2005)

Common marker	AA map	Y13Zh	Y13Fu	Y13JII	Composite map
TC9B08	Group 1	Lg2			LG2
TC3H02	Group 1	Lg5	Lg13		LG5
TC2D06	Group 1	Lg8	Lg8		LG8
TC4G05	Group 1	Lg8	Lg8		LG8
RN0 × 615	Group 2	Lg3	Lg3		LG3
RI1F06	Group 2	Lg3	Lg3		LG3
PM32	Group 2	Lg3	Lg3		LG3
TC4A02	Group 2	Lg7			LG7
TC11A02	Group 2	Lg7	Lg7		LG7
TC1G04	Group 2	Lg10	Lg10		LG10
TC4F12	Group 2	Lg11	Lg11		LG11
TC1E06	Group 3	Lg4			LG4
TC2A02	Group 3	Lg4	Lg12		LG4
TC3E02	Group 3	Lg4	Lg12		LG4
PM3	Group 3	Lg4	Lg4	Lg4	LG4
PM238	Group 3	Lg4			LG4
TC4E10	Group 3			Lg4	LG4
TC11E04	Group 3			Lg13	LG22
Gi-832	Group 4	Lg1			LG1
RN9C02	Group 4	Lg1			LG1
Ah-569	Group 4	Lg1			LG1
Ah-408	Group 4	Lg1			LG1
TC6E01	Group 5	Lg12	Lg5	Lg5	LG5
AH4-26	Group 5		Lg5		LG5
PM35	Group 5	Lg6		Lg6	LG6
TC1D02	Group 5	Lg14	Lg14		LG14
TC7H02	Group 5	Lg15			LG15
PM36	Group 5	Lg19	Lg19	Lg10	LG19
TC3H07	Group 6	Lg5	Lg21		LG5
AC2H11	Group 6	Lg5	Lg21		LG5
TC11A04	Group 6	Lg6			LG6
TC1A02	Group 6	Lg6			LG6
TC7C06	Group 6	Lg6			LG6
TC1A08	Group 6	Lg15	Lg15		LG15
TC5A06	Group 6	Lg18		Lg9	LG18
TC4G10	Group 7	Lg9	Lg9		LG9
TC9H08	Group 7	Lg17	Lg17	Lg3	LG17
PM204	Group 7		Lg17	Lg3	LG17
Gi-716	Group 8	Lg2	Lg2	Lg1	LG2
TC1E05	Group 8	Lg2			LG2
TC6H03	Group 8	Lg2	Lg2		LG2
TC9F10	Group 8	Lg2	Lg2		LG2
TC9F04	Group 8	Lg3	Lg3		LG3
PM188	Group 8	Lg16	Lg16		LG16
TC3B04	Group 11	Lg2	Lg2	Lg2	LG2
TC7A02	Group 11	Lg2	Lg2		LG2
TC3B05	Group 11	Lg2			LG2

## Discussion

A composite linkage map was constructed from three individual RIL mapping populations with 175 SSR markers on 22 composite linkage groups. The three RIL mapping populations, Y13Zh, Y13Fu and Y13J11, were derived from crosses using a common female parent Y13, a Spanish bunch type with high yield. The male parents were a Virginia type with dark purple testa and high protein content (32.4%), a Spanish type with high oil content (56.2%), and a Spanish type with reported resistance to *A. flavus *and aflatoxin contamination [31-33]. Therefore, these RIL populations could be used for QTL studies for several important traits. This map also consists of 47 SSRs that have been used in the published AA genome map [13] and could be used in comparative mapping studies. The important contributions are the collected 1044 SSR primer pairs provided as an additional files, and the three RIL populations are also made available to public upon request for QTL analysis and linkage map improvement.

### The composite genetic linkage map length and segregation distortion

The composite genetic linkage map covers 885.4 cM and comprises 175 loci distributed over 22 linkage groups. Seven of the linkage groups include only 2 or 3 markers, which could have resulted from the low density of markers. These small linkage groups could be artificial and additional genetic markers are needed to improve the linkage analysis and the assignment. The composite map distance was much shorter than the one of the synthetic tetraploid map with RFLP markers (map distance = 2210 cM) [12] and the two maps of the AA genome (1063 cM and 1230.89 cM) [11,13]. Several factors could account for the reduced length of the composite map compared to the other three maps in *Arachis*. The first factor could be that the markers on the composite map were far from saturated. The 175 markers distributed on the map were much fewer than the synthetic tetraploid map which consisted of 370 RFLP markers. The second factor could be the different mapping software used in the linkage analysis. In general, maps constructed with JoinMap are shorter than those constructed with a multilocus-likelihood package such as Mapmaker or OUTMAP [34-36].

Marker distance and linkage group lengths were consistently larger with Mapmaker than JoinMap, even using the same mapping function (Kosambi) [37]. For comparison we also constructed the linkage groups for population Y13Fu using Mapmaker (Additional files 3 and 4). The results also showed that the linkage groups constructed with JoinMap (540.7 cM) were much shorter than those constructed with Mapmaker (694.6 cM). The multilocus-likelihood method used by Mapmaker assumes an absence of crossover interference; when interference is present, JoinMap correctly produces shorter maps, even though both programs use the Kosambi mapping function [38]. This difference was also observed in other studies [39,40].

The segregation distortion was 22.8%, 13.6% and 8.5% of the polymorphic markers in these three populations. A total of 93 markers were in common in at least two of the three mapping populations. Seventeen markers were genotyped in all three populations. In addition, there are 68 more common markers between populations Y13Zh and Y13Fu; 5 more markers between populations Y13Zh and Y13J11, and 3 more SSR markers between populations Y13Fu and Y13J11. Based on the common markers and the comparison between individual maps, the majority of the linkage groups were consistent among the individual maps with few exceptions (Additional file 2). For example, the 7 markers on Lg4 (Figure 1) in the population Y13Zh were placed on two Lgs in the population Y13Fu (5 markers on Lg4 and 2 markers on Lg12) (Figure 2). The markers on Lg5 in population Y13Zh (Figure 1) distributed on 3 Lgs (Lg5, Lg13, and Lg21) in population Y13Fu (Figure 2). The listed inconsistencies may be due to the low density of this linkage map as showing the markers derived from the same linkage group in one population were assigned into different linkage groups in another population (Additional files 3 and 4).

Segregation distortion has been reported and the reasons for distortion of segregation ratios may be due to the factors such as chromosome loss [41], genetic isolation mechanisms [42], and the presence of viability genes [43,44]. Non biological factors such as scoring errors [45,46] and sampling errors [47,48] can also lead to distortion in segregation ratios. The proportions of distorted markers in population Y13Zh is higher (23.0%) than in the population Y13Fu (13.5%) and population Y13J11 (7.8%). Both biological and not biological factors could cause the observed segregation distortion in these populations.

### Comparison of the composite tetraploid map and the AA diploid map

On this tetraploid map there are 47 SSRs from the AA diploid wild peanut (*A. duranensis *× *A. stenosperm*a) [13]. Therefore, these SSR markers could provide a set of easily shared markers that can be used to cross reference the tetraploid map to SSR-based AA genome map. On the basis of these common SSR markers, it could determine synteny between cultivated peanut and wild progenitor. The primary goal for the construction of this composite map was to place, relative to one another, as many SSR markers as possible onto a single map. Therefore, the concern is more towards obtaining a general order and distance among these makers rather than the fine resolution of order and distance. With 175 SSR markers, this map will be a useful resource and a reference map, in which markers may be selected for future mapping projects within *A. hypogaea *and for comparative studies among other *Arachis *species. For example, combining information from multiple pedigrees is necessary if important traits do not segregate within a single population. Often, it is not practical or necessary to construct complete genetic maps to identify the genomic location of the traits. However, such studies can still be related to the entire genome by selecting markers suitable for superimposing the detailed region onto the composite map. In comparison with the SSR-based AA genome map [13] there are agreements in the composite map to the AA map, such as LG4 and Group 3. The comparative map will provide interesting information in genomic structure analysis and the relationship between the diploid wild species and the tetraploid cultivated peanuts. This SSR-based tetraploid reference map provides a framework and represents an ideal starting point for future mapping projects in *Arachis *since the stable and transferable SSR makers of the map can be saturated with other types of makers such as SNP and integrated into a consensus enhanced-density tetraploid map for *Arachis *in the future.

### Low polymorphism of EST-SSR

Only 4.3%, 2.3% and 0.3% of EST-SSRs produced useful polymorphic makers in these populations. In contrast, when genomic DNA sequences were used as the source of SSR-containing sequences, 23.6%, 20.9% and 11.4% yielded markers that were polymorphic in these populations. The possible explanation is that markers derived from genomic sequences contained more repeat units as well as a greater range of allele sizes and genetic diversity than markers isolated from EST libraries. The striking difference of polymorphism between the peanut SSRs derived from the two sources is consistent with differences reported in other crops. For example, Arshchenkova and Ganal [49] reported that only 20 of 27,000 tomato ESTs contained SSRs of more than ten repeat units. In barley EST-derived SSRs were generally shorter (7.3 repeat units) than genomic DNA-derived SSRs (22.7 repeat units) [50]. The average number of repeats from EST-derived and genomic DNA-derived SSRs was 6.1 versus 13.7 in sugarcane [51]. Smulders *et al*. [52] reported that SSR markers derived from fewer repeats were reported to be significantly less polymorphic than markers generated from longer repeats. Other factors such as selection against large alterations in coding regions and associated sequences that may play a role in gene expression could constrain SSR expansion or contraction. Such constraints could contribute to the reduced polymorphism of EST-SSRs.

## Conclusions

This manuscript reports the construction of a SSR-based map of *A. hypogaea*. The primary goal for the construction of this map was to develop a framework for future improvement. With 175 SSR markers, this map will be a useful resource and tool, in which SSR markers may be used for future mapping projects. In comparison with the SSR-based AA diploid map, clearly there is homologus or synteny between LG4 of tetraploid map and Group 3 of AA diploid map. This SSR-based tetraploid map provides a valuable genetic framework for qualitative and quantitative trait analysis in *A. hypogaea*. In addition, most of the primer sequences of SSR markers used in construction of current map were derived from the original papers. Thus, researchers from different laboratories can use these markers for map development and comparative mapping. All the markers used in this study have been made available as additional files and the RILs will be available to collaborators upon request. In concert with other maps in progress, this reference map represents an ideal starting point and provides a framework since the stable and transferable SSR makers can be saturated with other types of makers such as SNP. We could develop a consensus enhanced-density tetraploid map for *A. hypogaea*.

## Methods

### Population construction

Three RIL (recombinant inbred line) populations were constructed from three crosses. Yueyou 13 (Y13), a Spanish type with high yield, was the common female parent in all three populations. The male parents for the populations were Zhenzhuhei (population Y13Zh), a Virginia type with dark purple testa and high protein (32.4%) content, Fu 95-5 (population Y13Fu), a Spanish type with high oil content (56.2%), and J11 (population Y13J11), a Spanish type with reported resistance to *Aspergillus flavus *and aflatoxin contamination [31-33]. The populations were advanced to the F_4 _by single seed descent. Individual plants were harvested and progeny rows were grown to produce the F_4:6 _RIL populations. The populations consisted of 142 individual lines for Y13Zh, 84 individual lines for Y13Fu, and 136 individual lines for Y13J11. Total genomic DNA was extracted from young leaves of peanuts according the protocol used by Moretzsohn *et al*. [13].

### SSR markers and screening for polymorphism

SSR markers in this study were primarily collected from published data, such as SSR-enriched genomic sequences, expressed sequence tags (ESTs), and by "data mining" peanut EST sequences in GenBank by searching for the presence of di-, tri-, tetra- and pentanucleotide repeat motifs. A total of 1044 primer pairs were selected, including 170 SSRs previously mapped in the diploid wild peanut [13]. There were 652 SSR markers derived from genomic DNA and 392 from ESTs (refer to Additional file 1).

Forward and reverse primers were synthesized by Sigma-Genosys (Woodlands, TX). All primer pairs were screened against the parental lines for polymorphism. The polymorphic markers were then used to genotype individual RILs of each population. PCR was performed in 96-well plates in MJ Research PTC200 thermocycler (Waltham, MA). PCR reactions were prepared in a volume of 12.5 μl containing 10× Taq polymerase buffer (500 mM KCl, 100 mM Tris- HCI pH 8.5, and 1 mg/ml gelatin), 1.0 mM MgCl_2_, 0.5 mM dNTPs, 5 pmol of each primer, 0.25 U *Taq *polymerase, and 25 ng of template DNA. The final volume was adjusted with sterile distilled water. The PCR amplifications were conditioned as follows: 95°C for 10 min, followed by 30 cycles at 95°C for 1 min, a specific annealing temperature of a specific primer pair for 1 min and 72°C for 1 min, and the final extension was 72°C for 10 min. The specific annealing temperatures were from 54°C to 60°C. The PCR products were separated on 6% non-denaturing polyacrylamide gels (PAGE) and visualized by silver staining.

### Linkage map construction

SSR markers consolidated in this study are available as an electronic additional file, including SSR name, forward and reverse primer sequences, and polymorphism or monomorphism in each population. Each marker was scored individually for each line and compiled into a single excel file based on parental segregation data. Segregation patterns were assigned to each marker by following JoinMap data entry notation (<aa × bb>). Linkage analysis was performed with JoinMap 3.0 [30], which analyzes cross-pollinated populations derived from homologous parents to create an individual linkage map. The "locus genotype frequency" function calculated chi-square values for each marker to test for expected 1:1 segregation ratio. Markers were placed into linkage groups with the "LOD groupings" and "create groups for mapping" command using the Kosambi map function [37]. Calculation parameters were set for a minimum LOD of 3 and recombination fraction of 0.45. Marker order in groups was established using the "Calculate Map" command. Linkage groups with common markers on individual maps were merged to create a composite map using "Join-combine groups for map integration" command. Linkage maps were drawn using MapChart for Windows [53].

## Authors' contributions

YBH performed genotyping, data collection and linkage map construction, and prepared the first draft of the manuscript. XPC performed the sequence search and EST-SSRs. XQL oversaw the project and mapping population development. HYL was responsible for SSR screening. GYZ developed the RIL populations. SXL did DNA extraction. SJW conducted RIL genotyping. CCH participated in EST sequencing and revised the manuscript. BZG conceived and completed the research of EST sequencing, and revised and submitted the manuscript. All authors read and approved the final manuscript.

## Supplementary Material

Additional file 1 **All SSR marker information**. All SSR markers used in this study and polymorphisms among these populations. Click here for file

Additional file 2 **Comparative map information**. Comparison of individual maps and the composite map. Click here for file

Additional file 3 **Comparative information between JoinMap and Mapmaker**. Comparison of Y13Fu Maps constructed by JoinMap and Mapmaker. Click here for file

Additional file 4 **Y13Fu linkage map by Mapmaker**. Population Y13Fu linkage groups by Mapmaker. Click here for file
