# Supplementary material for: A SSR-based composite genetic linkage map for the cultivated peanut (Arachis hypogaea L.) genome
Source: BMC Plant Biol. 2010 Jan 27;10:17. doi: 10.1186/1471-2229-10-17 (PMC2835713; doi:10.1186/1471-2229-10-17)
Supplement: Supplementary file 1 [file 1471-2229-10-17-S4.DOC]

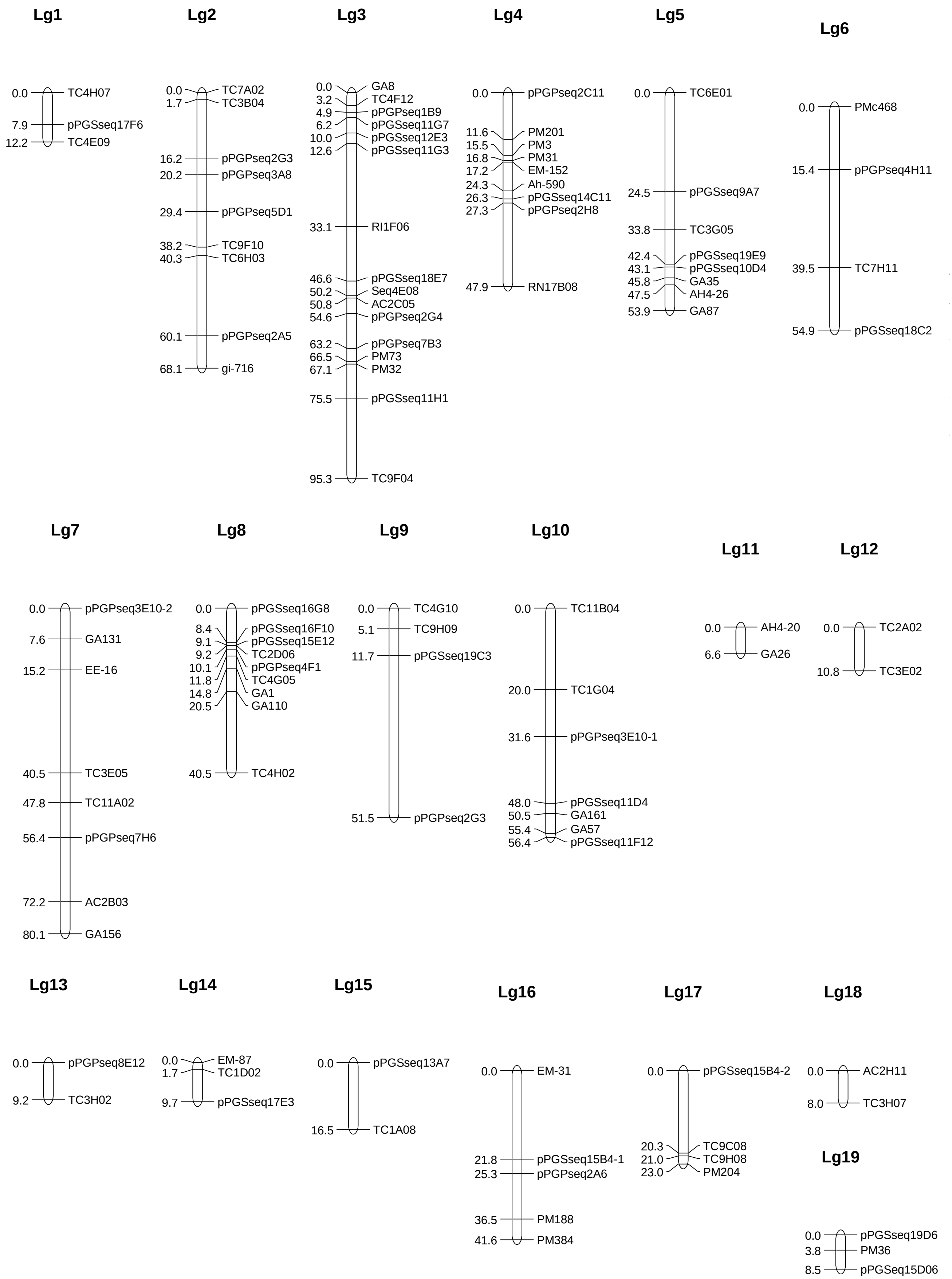
Lg1
0.0 TC4H07
7.9 pPGSseq17F6
12.2 TC4E09
Lg2
0.0 TC7A02
1.7 TC3B04
16.2 pPGPseq2G3
20.2 pPGPseq3A8
29.4 pPGPseq5D1
38.2 TC9F10
40.3 TC6H03
60.1 pPGPseq2A5
68.1 gi-716
Lg3
0.0 GA8
3.2 TC4F12
4.9 pPGPseq1B9
6.2 pPGSseq11G7
10.0 pPGSseq12E3
12.6 pPGSseq11G3
33.1 RI1F06
46.6 pPGSseq18E7
50.2 Seq4E08
50.8 AC2C05
54.6 pPGPseq2G4
63.2 pPGPseq7B3
66.5 PM73
67.1 PM32
75.5 pPGSseq11H1
95.3 TC9F04
Lg4
0.0 pPGPseq2C11
11.6 PM201
15.5 PM3
16.8 PM31
17.2 EM-152
24.3 Ah-590
26.3 pPGSseq14C11
27.3 pPGPseq2H8
47.9 RN17B08
Lg5
0.0 TC6E01
24.5 pPGSseq9A7
33.8 TC3G05
42.4 pPGSseq19E9
43.1 pPGSseq10D4
45.8 GA35
47.5 AH4-26
53.9 GA87
Lg6
0.0 PMc468
15.4 pPGPseq4H11
39.5 TC7H11
54.9 pPGSseq18C2
Lg7
0.0 pPGPseq3E10-2
7.6 GA131
15.2 EE-16
40.5 TC3E05
47.8 TC11A02
56.4 pPGPseq7H6
72.2 AC2B03
80.1 GA156
Lg8
0.0 pPGSseq16G8
8.4 pPGSseq16F10
9.1 pPGSseq15E12
9.2 TC2D06
10.1 pPGPseq4F1
11.8 TC4G05
14.8 GA1
20.5 GA110
40.5 TC4H02
Lg9
0.0 TC4G10
5.1 TC9H09
11.7 pPGSseq19C3
51.5 pPGPseq2G3
Lg10
0.0 TC11B04
20.0 TC1G04
31.6 pPGPseq3E10-1
48.0 pPGSseq11D4
50.5 GA161
55.4 GA57
56.4 pPGSseq11F12
Lg11
0.0 AH4-20
6.6 GA26
Lg12
0.0 TC2A02
10.8 TC3E02
Lg13
0.0 pPGPseq8E12
9.2 TC3H02
Lg14
0.0 EM-87
1.7 TC1D02
9.7 pPGSseq17E3
Lg15
0.0 pPGSseq13A7
16.5 TC1A08
Lg16
0.0 EM-31
21.8 pPGSseq15B4-1
25.3 pPGPseq2A6
36.5 PM188
41.6 PM384
Lg17
0.0 pPGSseq15B4-2
20.3 TC9C08
21.0 TC9H08
23.0 PM204
Lg18
0.0 AC2H11
8.0 TC3H07
Lg19
0.0 pPGSseq19D6
3.8 PM36
8.5 pPGSeq15D06
